# A case of treatable hypertension: fibromuscular dysplasia of renal arteries

**DOI:** 10.1186/s13104-015-1835-z

**Published:** 2016-01-02

**Authors:** Dissanayake Mudiyanselage Priyantha Udaya Kumara Ralapanawa, Kushalee Poornima Jayawickreme, Ekanayake Mudiyanselage Madhushanka Ekanayake

**Affiliations:** Department of Medicine, University of Peradeniya, Peradeniya, Sri Lanka

**Keywords:** Renovascular hypertension, Renal artery stenosis, Fibromuscular dysplasia, Angioplasty, Sri Lanka

## Abstract

**Background:**

Renovascular hypertension accounts for 51–52 % of all cases of hypertension in the general population, but plays a major role in treatable causes for hypertension in the young. This entity consists of renal vascular atherosclerosis (90 %), commonly seen among the elderly population, and renal fibro muscular dysplasia (FMD) (10 %), predominantly seen in the young. The prevalence of clinically significant renal artery fibromuscular dysplasia is 0.4 %.

**Case presentation:**

We present a case of treatable young hypertension in a 29 year old female, who was diagnosed with renovascular hypertension due to fibromuscular dysplasia of the left renal artery. Computed tomographic angiogram revealed significant stenosis of the left main renal artery. Diethylene triamine penta acetic acid renogram showed a small left kidney due to renal artery stenosis. She underwent left sided nephrectomy, and histology revealed features of FMD, after which she achieved full recovery with normalization of blood pressure, and did not require antihypertensive drug treatment.

**Conclusions:**

Fibromuscular dysplasia causing renal artery stenosis, though a rare cause of renovascular hypertension, is essential to be considered in young hypertensives, even in the absence of family history of hypertension. A high index of suspicion is necessary in early diagnosis and prompt treatment, which can result in rapid and complete recovery.

## Background

Renovascular hypertension is due to renal artery stenosis (RAS) leading to reduced renal perfusion activating the renin angiotensin aldosterone system, resulting in hypertension [1]. It accounts for 1–2 % of all cases of hypertension in the general population [2], and 5.8 % of secondary hypertension [3], but plays a major role in completely treatable causes of hypertension in the young. Renovascular hypertension is characterized by features like hypokalemia, young age of onset, and renal bruit [1]. This entity consists of renal vascular atherosclerosis and fibromuscular dysplasia (FMD). Atherosclerosis; accounting for 90 % of RAS, is seen in the elderly and in those with high cardiovascular risk factors. Whereas FMD accounts for less than 10 % of RAS, as is seen in females between 15 and 50 years [1]. FMD is a non-atherosclerotic, non-inflammatory angiopathy which could affect any vascular bed, but mainly affects renal arteries [4]. Other rarer cause of RAS include aortitis, radiation induced arteritis, dissecting aneurysm and von-Recklinghausen’s disease. In this paper, we present a case of successfully treated young hypertension due to RAS caused by FMD.

## Case presentation

A 29 year old Sinhalese Sri Lankan female, who was apparently well, presented with incidentally detected high blood pressure. She is a mother of two children, but had no history of pregnancy induced hypertension. She denied any family history of hypertension. She also complained of loss of appetite and subjective weight loss during the past few months.

Her physical examination revealed a blood pressure of 180/120 mmHg on two separate occasions, and was equal in both arms. Her pulse rate was 88 beats per minute, with no radio-radial or radio-femoral delay. There were no renal masses, and no carotid, renal or femoral artery bruits. Her cardiovascular and central nervous system examination was unremarkable, and had no evidence of retinopathy on fundoscopy. She had no peripheral stigmata of atherosclerosis, or endocrinopathies.

Her renal function tests, serum electrolytes, urine full report, full blood count, erythrocyte sedimentation rate, and liver function tests were all normal. Her electrocardiogram and transthoracic echocardiogram were unremarkable.

**Fig. 1:**
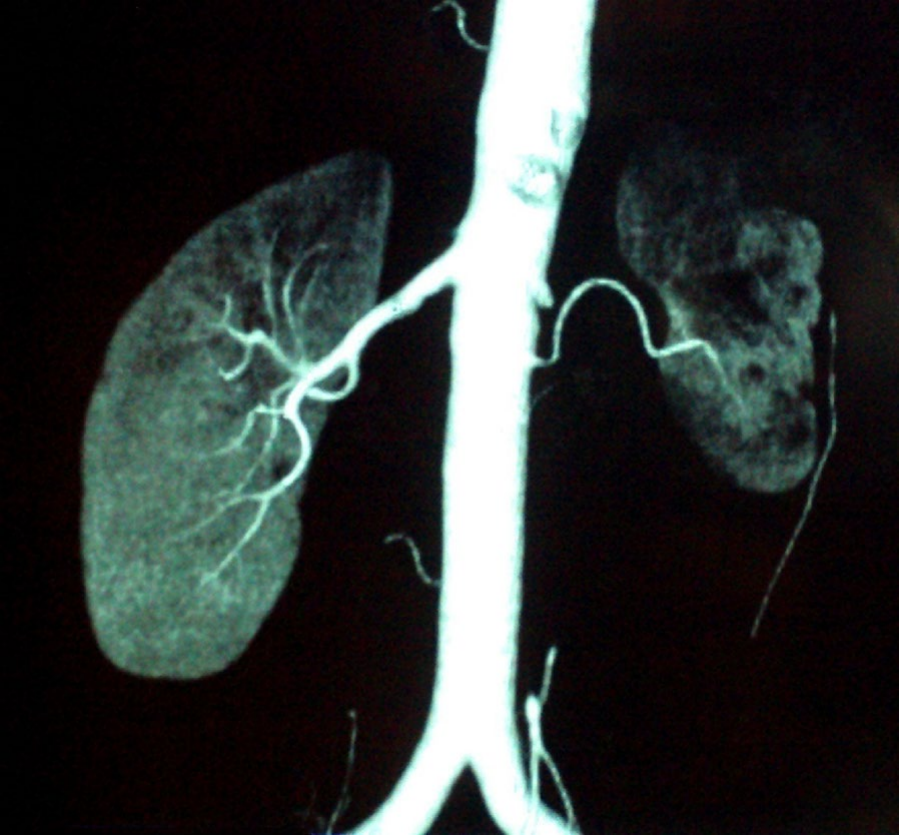
Close-up of CT angiogram showing significant left sided renal artery stenosis, with an atrophied left kidney, which is supplied by an accessory renal artery. There is normal perfusion and function of the right kidney

**Fig. 2:**
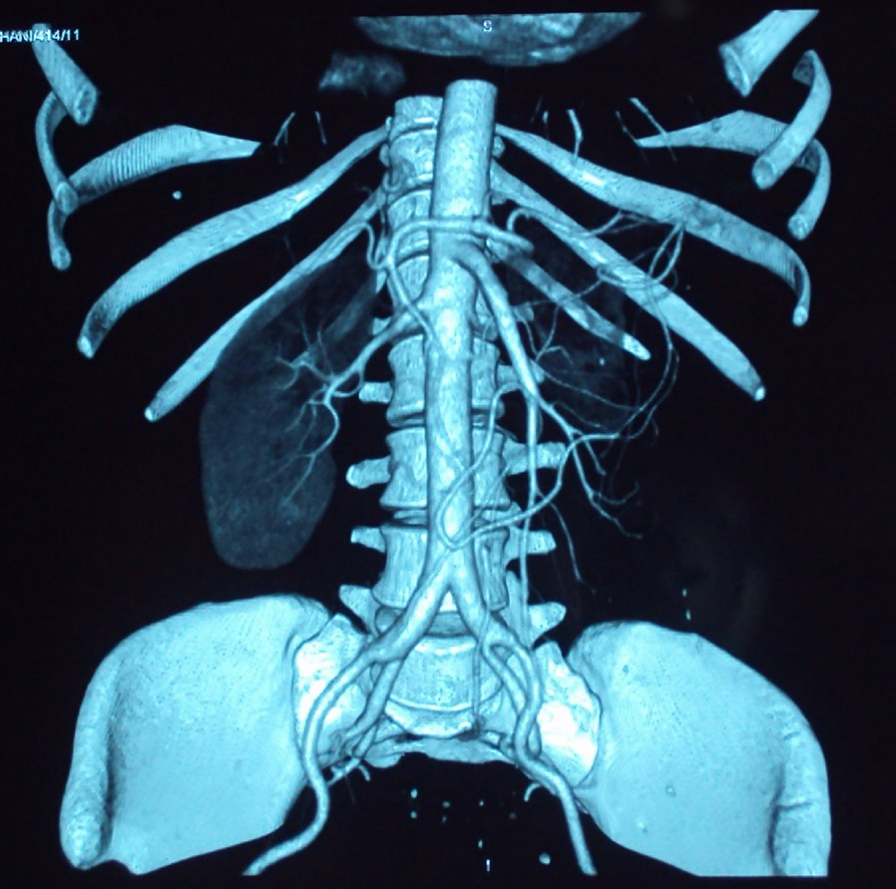
CT renal angiogram showing atrophic left kidney with non visualization of the left main renal artery

As she was a recent onset young hypertensive, she was investigated with ultrasound scan of the abdomen which showed non visualization of the left kidney. Therefore, computed tomography angiogram (CTA) and diethylene triamine penta acetic acid (DTPA) renogram was indicated. DTPA renogram showed a small left kidney which was suggested to be either congenital, or due to RAS, with normal perfusion and function of the right kidney. CTA revealed significant stenosis of the left main renal artery, which was suggested to be due to FMD, and an accessory renal artery supplying the lower pole of the left kidney was detected (Figs. 1, 2). After evaluation of her renovascular hypertension, she was referred to a vascular surgeon and underwent left sided nephrectomy, and histology revealed features of FMD of left renal artery. She achieved full recovery with normalization of blood pressure following surgery, and is currently not on any antihypertensive medication.

## Discussion

FMD is an uncommon angiopathy that predominantly affects young to middle-aged females [5], which is non-atherosclerotic, and non-inflammatory, and most commonly affecting the renal and internal carotid arteries, but may be seen in any arterial bed [4]. A pathological classification of renal artery FMD was proposed by McCormack et al. [6] and revised by Stanley [7]. Three main subtypes were identified based on the dominant arterial wall layer involved, namely; intimal, medial, and adventitial (perimedial) FMD. Intimal, medial, and perimedial FMD accounts for 5, 85, and 10 % cases of renal artery FMD respectively [2]. A study done by *Alimi* et al found that 66 % of cases had more than one arterial layer involved [8]. Aneurysms and dissections are considered to be complications of FMD [7].

A definite etiology of FMD is not known, though there are various theories. Genetic predisposition is proposed, as a study done by *Rushton* showed 60 % cases to have autosomal dominant inheritance pattern with variable penetrance [9], and a subsequent case report of disease among family members also support this theory [10]. Other proposed mechanisms of etiology are hormonal factors, mechanical trauma, metabolic and immunologic factors, and intrinsic deficiency of elastic fibers [11]. There is evidence that cigarette smoking may be a risk factor [12]. FMD is also associated with pheochromocytoma, neurofibromatosis, Ehlers-Danlos syndrome type IV, Marfan’s syndrome, Alport’s syndrome, and Takayasu’s arteritis [4]. Atherosclerosis, which is its main differential diagnosis, is differentiated by being located at the ostium or proximal portion of the artery in older patients with typical cardiovascular risk factors, where as FMD occurs in the middle or distal arterial segments in younger patients with few cardiovascular risk factors [4]. Unlike RAS due to atherosclerosis, FMD rarely has deterioration of renal function with high serum creatinine levels [2]. The other differential diagnosis is Polyarteritis nodosa which shows pathognomonic multiple focal aneurysms on renal angiography [13].

FMD accounts for 10–20 % of documented RAS, with renovascular hypertension accounting for 1–2 % of hypertensives, and the prevalence of clinically significant renal artery FMD can be estimated to be about 0.4 % [2]. The commonest arterial involvement of FMD is of renal arteries (60–75 %), followed by cervico-cranial arteries with a prevalence of half that of renal arterial involvement [14], with at least two vascular beds involved in up to 28 % cases [15]. The clinical presentation may vary from an asymptomatic condition to a multisystem disease depending on the arterial segment involved, the degree of stenosis, and the type of FMD.

The commonest presentation of FMD is renovascular hypertention; usually grade 2–3, or resistant hypertension. The mechanism depends on whether the stenosis is unilateral (renin-dependent hypertension) or bilateral/unilateral with a single functioning kidney (volume-mediated hypertension) [16]. However 2/3 RAS due to FMD are bilateral [17]. Ultimately there is activation of the renin angiotensin aldosterone system resulting in vasoconstriction, and salt and water retention. FMD may be complicated by renal artery dissection and kidney infarction with abrupt flank pain, haematuria and rapidly progressive hypertension [18].

Various imaging methods are used in the evaluation of RAS. Duplex imaging of the renal arteries can accurately detect elevated blood-flow velocities in the proximal and distal portions of these arteries, but has a 10–20 % failure rate due to operator’s inexperience, the presence of obesity or bowel gas, respiratory renal movements, and the fact that the possibility of a stenotic accessory renal artery cannot be excluded by visualization of a single normal renal artery [19]. The current importance of ultrasound scan is the ability to predict functional recovery based on the measurement of resistive index. Captopril renography (DTPA renography) is a safe noninvasive tool used in the screening for RAS, but data regarding its reliability is inconsistent. Its efficacy is increased by administering 25–50 mg of captopril 1 h prior to injection of radioisotope, and the sensitivity and specificity decreases in the presence of azotemia, bilateral disease, or disease of solitary functional kidney [20]. Computed tomographic angiography is the most specific tool in diagnosis, where as gadolinium enhanced magnetic resonance angiography has the additional advantage of not having radiation exposure, and limited nephrotoxicity. However both modalities have high specificity, but low sensitivity [21]. The gold standard in diagnosing renal artery FMD is intra-arterial angiogram with digital substraction, but is reserved for patients for whom it is clinically justified to proceed with revascularization in the same procedure, as it is an invasive test. Multifocal stenosis with the “string of beads” appearance is characteristic of FMD, which likely indicates the presence of medial type FMD, where as other features are tubular or focal lesions [22].

Pharmacological treatment of hypertension in FMD should follow the guidelines of the Joint National Committee on prevention, detection, evaluation and treatment of high blood pressure [23]. Almost all patients with RAS require at least one antihypertensive agent, and require three or more medications twice as frequently as those with cerebrovascular FMD [24]. Revascularization is the choice of treatment in patients with young hypertension refractory to pharmacological therapy, those who are intolerant to antihypertensive, those who have lost renal volume due to ischaemic nephropathy, and the goal is to cure the disease [4]. Surgical revascularization was the primary treatment modality before the introduction of percutaneous transluminal angioplasty techniques (PTA), and leads to improvement of hypertension in 60–88 % cases [24, 25]. Nowadays PTA has emerged as a mainstay of treatment for patients with FMD who meet the criteria for interventions, and leads to improvement or resolution of hypertension in 60–80 % of cases [24, 25]. It is less costly than surgical revascularization and less invasive, and can be performed on an outpatient basis and has a lower morbidity [4]. Complications of percutaneous interventions occur in 14 % of cases and usually minute but rarely renal artery perforation, dissection or segmental renal infarctions may occur [27–29]. Successfully performed renal angioplasty results in substantial and rapid reduction in both systolic and diastolic blood pressure to normal values and it correlates with a marked reduction in plasma renin activity and angiotensin II levels [4, 30]. Complete resolution of hypertension without the requirement of antihypertensive medication may be achieved only in 30–50 % cases [24, 25]. Imaging should be performed soon after revascularization process to assess the adequacy of the intervention [31], again in 6 months, 12 months and yearly there after [3]. When the criteria for revascularization are not met, or in extreme hypertension, or following failed primary surgery, or when a kidney is non-viable nephrectomy can be performed as in this case, resulting in complete cure [25].

## Conclusions

FMD causing RAS, though a rare cause of renovascular hypertension is essential to be considered in young hypertensives, even in the absence of family history of hypertension. A high index of suspicion is necessary in early diagnosis and prompt treatment, which would result in rapid and complete recovery.

## Consent

Written informed consent was obtained from the patient for publication of this case report and accompanying images.

## Abbreviations

CTA: computed tomography angiogram
DTPA: diethylene triamine penta acetic acid
FMD: fibro muscular dysplasia
RAS: renal artery stenosis
PTA: percutaneous transluminal angioplasty
